# Responses of Biogeochemical Characteristics and Enzyme Activities in Sediment to Climate Warming under a Simulation Experiment in Geographically Isolated Wetlands of the Hulunbuir Grassland, China

**DOI:** 10.3390/ijerph14090968

**Published:** 2017-08-27

**Authors:** Liliang Han, Derong Su, Shihai Lv, Yan Luo, Xingfu Li, Jian Jiao, Zhaoyan Diao, He Bu

**Affiliations:** 1 Research Center for Grassland Ecology and Resources, Beijing Forestry University, Beijing 100083, China; dapao1918@bjfu.edu.cn (L.H.); Luoyabc@163.com (Y.L.); lixingfu0@163.com (X.L.); jiaojian@bjfu.edu.cn (J.J.); 2 State Environmental Protection Key Laboratory of Regional Eco-process and Function Assessment, State Key Laboratory of Environmental Criteria and Risk Assessment, Chinese Research Academy of Environmental Sciences (CRAES), Beijing 100012, China; lvsh1963@163.com (S.L.); diaozy@126.com (Z.D.); 3 Hui River National Nature Reserve Administration of Inner Mongolia, Hailar 021100, Inner Mongolia, China; hhnre@126.com

**Keywords:** geographically-isolated wetland, climate warming, biogeochemical characteristic, enzyme activity, sediment

## Abstract

Climate warming generates a tremendous threat to the stability of geographically-isolated wetland (GIW) ecosystems and changes the type of evaporation and atmospheric precipitation in a region. The intrinsic balance of biogeochemical processes and enzyme activity in GIWs may be altered as well. In this paper, we sampled three types of GIWs exhibiting different kinds of flooding periods. With the participation of real-time temperature regulation measures, we assembled a computer-mediated wetland warming micro-system in June 2016 to simulate climate situation of ambient temperature (control group) and two experimental temperature differences (+2.5 °C and +5.0 °C) following a scientific climate change circumstance based on daily and monthly temperature monitoring at a two-minutes scale. Our results demonstrate that the contents of the total organic carbon (TOC), total nitrogen (TN), and total phosphorus (TP) in the warmed showed, roughly, a balance or a slight decrease than the control treatment. Warming obstructed the natural subsidence of sediment, but reinforced the character of the ecological source, and reduced the activity of urease (URE), but promoted the activity of alkaline phosphatase (AKP) and sucrase (SUC). Redundancy analysis showed that sucrase, urease, available phosphorus (AP), and pH were the major correlating factors under warming conditions in our research scope. Total organic carbon, total nitrogen, sucrase, catalase (CAT), and alkaline phosphatase were the principal reference factors to reflect the ambient temperature variations. Nutrient compositions and enzyme activities in GIW ecosystems could be reconstructed under the warming influence.

## 1. Introduction

“Geographically isolated wetlands” (GIWs) are a diverse wetland type, which lack surface water connections to surrounding water bodies and are generally defined as entirely surrounded by uplands [1]. GIW has ecological and intermittent surficial hydrology connectivity, geo-hydrologic connectivity [2], determining adjacency and, subsequently, relative isolation [3,4], as well as biogeochemical connectivity [5]. GIWs’ soil moisture content varies greatly, comparatively, and lacks a connection with surface water in normal conditions. GIWs can be very important carbon storage sinks that will drastically alleviate environmental pressures from climate change impacts [6,7]. GIWs have a more sensitive response to water body fluctuation than rivers and lakes and, to some degree, regarded as an indicator for wetland degradation. The biogeochemical features and enzyme activities of GIWs may be affected significantly by regional climate and water conditions, such as the flooding period. The challenges isolated wetlands face grow large owing to environmental change. Furthermore, global land average surface temperature is estimated to increase by 1.4–5.8 °C by the end of 21st century [8]. Climate warming produces a tremendous threat to the stability of GIW ecosystems and changes the type of evaporation and atmospheric precipitation in the region. The intrinsic biogeochemical and water-salt balance in wetland ecosystems may be affected by climate warming [9,10,11].

Temperature has a remarkable effect on enzyme activity [12], and soil enzyme activity will be affected by climate warming in the course of changing carbon and nitrogen cycling, with respect to ecological processes significantly and subsequently. In addition, the activity of soil enzymes can demonstrate the ability of energy metabolism and soil nutrient mineralization in different ways [13,14,15]. Many researchers have investigated a whole train of temperature manipulation measures simulating the greenhouse effect to expound the influence of a temperature rise on biogeochemical properties and enzyme activities in wetland ecosystems. Studies have found that elevated water temperature has an obvious effect, showing marked differences in terms of nutrients within wetland pore-waters based on the model nutrient diffusive flux [16,17]. Some attempts include laboratory studies conducted to simulate the reactions of soil under warming conditions, such as mixed upper sediments incubated with a thermal gradient temperature bar [18,19], or using buried warming cables to heat land to simulate a heating effect [20]. The above-mentioned experiments provide a relatively novel perspective and reasonably workable means to demonstrate the characteristics and the interaction mechanism of biogeochemical properties influenced by temperature variation. Nevertheless, the simulated warming scenario in the studies discussed above was on account of scheduled temperature differences without containing sufficient actual daily and seasonally measurements in accumulated temperatures for warming treatments. Therefore, the simulation of experimental consequences may not scientifically indicate the realistic change trend of wetland ecosystems under the anticipated warming effect. Meanwhile, there is little published work on the influence of climate warming on enzyme activity and biogeochemical process in GIWs of grasslands.

In this study, six GIWs with different flooding periods were collected along the Hui River Basin in the Hulunbuir Grassland. A computer-mediated wetland warming micro-system was assembled in June 2016 with the participation of real-time temperature regulation measures to simulate a 2.5 °C and a 5.0 °C temperature difference compared to an ambient temperature control treatment following the scientific climate change circumstance-based, two minute-scale, daily and monthly temperature fluctuations. Our objective is to make detailed studies on the influence of the long-term warming effect on the biogeochemical cycle of sediment, determining the biogeochemical properties of four kinds of soil enzyme activities (sucrase, urease, alkaline phosphatase, and catalase). We adopt redundancy analysis (RDA) to explore the relation between biogeochemical properties and enzyme activity in sediment under a simulation of the warming circumstance. We attempt to better define the evaluation indicators for GIWs in different flooding period’s responses to climate warming.

## 2. Materials and Methods 

### 2.1. Micro-Environment Simulating Disposition 

**Figure 1:**
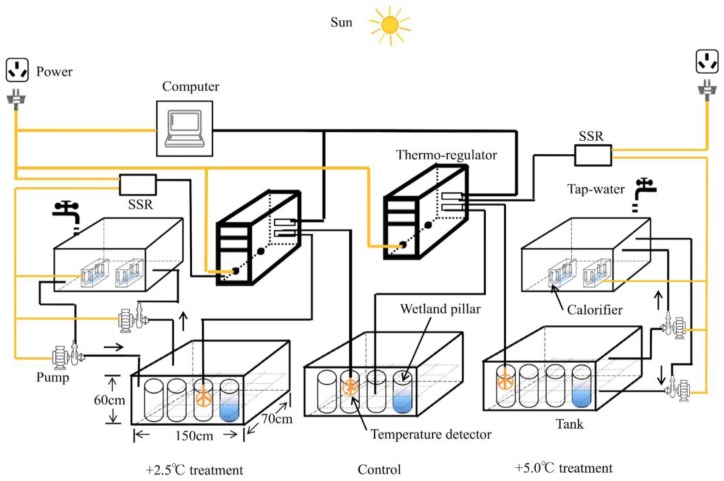
The experiment device of the simulation micro-environment under global warming conditions in geographically-isolated wetlands (GIWs) (left: control group +2.5 °C, low-warmed; middle: ambient water temperature, control; right: control group +5.0 °C, high-warmed).

An outdoor temperature sensor and monitoring device simulating climate change at a two-minute graduation for both daily and weekly scenes was operated by using a thermo-regulator and sensors independently for this study during two months. The device (Figure 1) consists of three major components: an intelligent temperature control and a record storage section, a sample storage appliance, and a water hydronic system. The sample storage appliance is composed of five plastic incubators: the intermediate position is for the current ambient temperature treatment (control), the left two boxes are for the 2.5 °C-increased temperature treatment (low-warmed), the right two cases are for the 5.0 °C-increased temperature treatment (high-warmed). The intelligent temperature control and record storage section is composed of customized control devices (XT-SM5-LCD, Xintai Microelectronics, Shenzhen, China), a solid-state relay (SSR, CDG1-1DA, DELIXI, Hangzhou, China) and a digital temperature detector (XT-2048, Beijing, China), a digital memory card (Kingston 32 GB Micro-SD, Kingston, CA, USA). The water hydronic system included a dual group of water pumps and calorifiers. The digital temperature detector, calorifier, and pumps were controlled by the home-made program (programmed in the C language). Under the control of the procedure, the immediate temperatures in the three wetland pillars which had no calorifier were recorded, and the overlying water temperature in the wetland pillars in both sides and that in the control treatment were contrasted by three digital temperature detectors constantly at a two-minute frequency. The two warmed groups were run by a 1.0 °C range of dipping and heaving, respectively. Henceforth, the homemade customized control device turned the pump and the heater on or off when the transient temperature difference was lower than 1.5 °C or over 3.5 °C in the low-warmed groups, but lower than 4 °C or over 6 °C in the high-warmed groups, respectively. All the components were placed outdoors for the entire experimental period. The simulator offers a realistic micro-environment viewpoint of varying patterns of isolated wetland biogeochemical characteristics and enzyme activities on different temperature gradient conditions, compared to the reported temperature modeling experiments.

### 2.2. Study Sites

**Figure 2:**
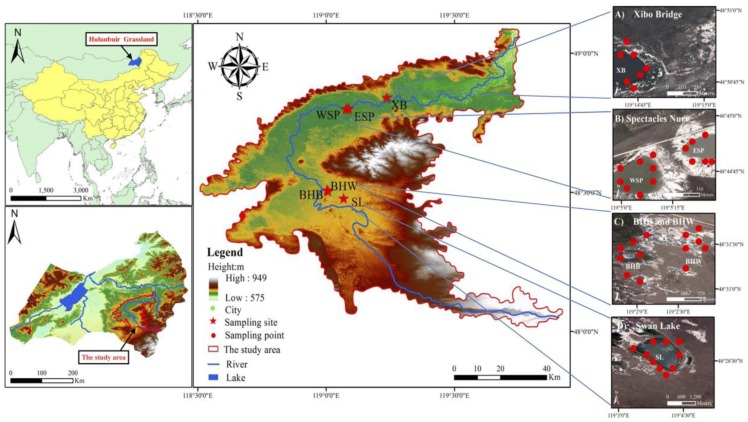
Location of sampling sites along the Hui River Basin of the Hulunbuir Grassland.

Sampling sites (Figure 2) are located in the middle and lower reaches of the Hui River, right in the east-central region of the Hulunbuir Grassland, northeastern China (118°46′48″–119°43′5″ E, 48°9′11″–49°0′7″ N), which belongs to the temperate continental climate zone. The annual average temperature is −2.4 °C to 2.2 °C with a frost-free period of 100–120 days. The annual precipitation in this area is 300–350 mm, of which more than 70% falls from June to August. The dominant families are Gramineae, Asteraceae, Rosaceae and Leguminosae. Geographically-isolated wetlands are prevalent in the study region across the immense Inner Mongolian Plateau. In June 2016, we selected six representative plots under different flooding periods from the geographically-isolated wetlands in the east-central region of the Hulunbuir Grassland in the Hui River Basin. The six sample plots are Swan Lake (SL), Xibo Bridge (XB), East Spectacles Pond (ESP), West Spectacles Pond (WSP), Bei Hui Black (BHB), and Bei Hui White (BHW). Among the sampling wetlands, SL presented a permanent flooding period, but XB, WSP, and ESP showed inter-annual periods, while BHB and BHW indicated seasonal periods.

### 2.3. Sampling Methods 

Opaque PVC pillars (55.0 cm height and 10.0 cm in internal diameter) were custom-made before sampling. Every single wetland pillar was designed to be filled with 30 cm of fresh sediment and 15 cm of the overlying water. Individual samples of the six sample plots’ sediment cores were collected to a depth of 30 cm from the surface with a stainless-steel sampler. These wetland core sediments (with three duplicates for each wetland site in all three treatment groups) were transported to the experimental station within two hours after collection and divided into two subsamples. One subsample of the fresh sediments was reserved for air drying, then ground homogenously and thoroughly, then passed through a 0.15 mm sieve for determining the original value of the sediment biogeochemical properties. The second part of the subsamples was transferred into the wetland pillar in the original structure after removing the larger stones, roots, and debris from the surface. After sediment filling in the blanks to a depth of 30 cm, each pillar was filled to a depth of 15 cm with overlying water from the corresponding sample site. Some of phytoplankton and aquatic vegetation was found growing normally after half a month in part of the wetland columns. Approximately 300 g of sediment was collected from each pillar using a sampler after 30 days and 60 days of incubation, respectively. In addition, the primary treatments of later column sediments were equal to the samples reserved earlier before the sediment was determined.

### 2.4. Sediment Biogeochemical Characteristics Analysis

Measurements of soil total organic carbon (TOC), total nitrogen (TN), ammonium nitrogen (NH_4_^+^-N), nitrate nitrogen (NO_3_^−^-N), total phosphorus (TP), available phosphorus (AP), pH, and electrical conductivity (EC) were taken on fresh sediment samples that were air-dried, crushed, and sieved, according to standard methods [21,22]. The soil total organic carbon (g∙kg^−1^) was determined using the potassium bichromate titrimetric method [23]. Sediment samples were analyzed for total nitrogen (TN) by Kjeldahl digestion [24]. Soil mineral N (NH_4_^+^-N and NO_3_^−^-N) was extracted using 2 mol·L^−1^ KCl solution. After extraction, NH_4_^+^-N was analyzed by the indophenol blue spectrophotometric method, and NO_3_^−^-N was determined by UV spectrophotometry at 220 and 275 nm. Calculating the difference between these two wavelengths readings can correct the disturbance from the dissolved organic matter. Total phosphorus in sediment was initially digested by mixed solution (concentrated sulfuric acid and perchloric acid), then phosphorus concentrations in the digested and extracted supernatant liquor were determined spectrophotometrically. Available phosphorus was extracted using the method reported in [25]. Sediment pH was measured using a pH electrode meter (pHS-3, Leici, Shanghai, China) in a 1:5 *v*/*v* sediment solution suspension. Soil electrical conductivity (EC) was determined using a conductivity meter (DDSJ-308F, Leici, Shanghai, China).

### 2.5. Enzyme Activity Assays

Activities of four sediment enzymes associated with carbon-cycling (sucrase), nitrogen-cycling (urease), phosphorus-cycling (alkaline phosphatase, and sediment respiration intensity (catalase) were determined. The traditional colorimetric methods were used for determining enzymes activities of wetland sediment. Enzyme assays test the latent activities of continuous and summative enzymes in sediments by inspecting the formation of products or the disappearance of substrates under optimum reactions [26]; for this reason, these assays demonstrate the relative number of enzymes in sediments.

The analytical methods of these enzymes were slightly modified from [27,28,29,30,31,32]. These enzymes’ activities, except catalase, were assayed with their appropriate substrate at their optimal acid-base environment and incubated at 37 °C for 24 h with an electro-heating standing-temperature cultivator. Subsequently, a developing agent was added into the extracted enzyme solutions for colorimetric analysis. Each assay contained a blank substrate with replenished deionized water but one sample blank was stetted with a lack of sediment in the whole assay, respectively. Absorbance was detected using a spectrophotometer (UV-2600, SHIMADZU, Kyoto, Japan). The slope of the linear regression between standard concentrations and absorbance (all *R*^2^ > 0.99) was used to calculate enzyme activity.

### 2.6. Statistical Analyses

Most of the statistical analyses were performed using SPSS (v. 20.0) for windows (SPSS Inc., Chicago, IL, USA). Redundancy analysis (RDA) was performed with CANOCO 4.5.1 (Microcomputer Power, Ithaca, NY, USA), and drawing by Origin Pro (v. 2015 SR2) (OriginLab, Northampton, MA, USA). The data collected from sediments during the experimental determination were given as mean ± standard error (S.E.) of three duplications. One-way analysis of variance (ANOVA) for the comparison of means was performed with SPSS software (v. 20.0) to analyze the significant differences of all six regions’ sediment original values and tested the effects of different heating treatments on control, low-warmed and high-warmed treatments. We used Pearson correlation analysis to study the significance of relationships between biogeochemical characteristics and enzyme activities. All statistical differences were determined using Duncan’s least significant differences (LSD) test at the 0.05 probability.

## 3. Results

### 3.1. Sediment Biogeochemical Properties

Table 1 presents the basic physicochemical properties of the six originally-managed geographically-isolated wetland sediment pillars. We can see that all the tested sediments were alkaline and the pH values indicated a narrow scope. The EC values varied greatly in the six sampling sites with different flooding period. At a regional scale, the sediments in SL indicated a higher content in TN, NO_3_^−^-N, SUC, and URE than the other five sampling site groups, obviously, as do the contents of AP and TP in BHB.

**Figure 3:**
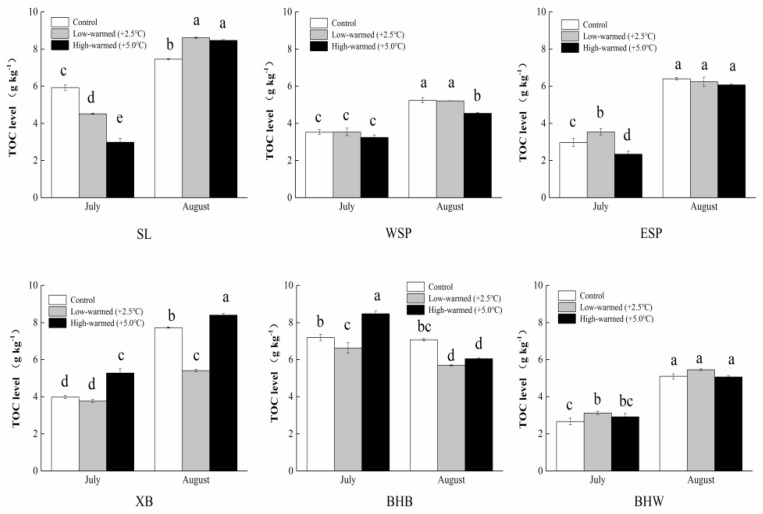
Contents of total organic carbon (TOC) in sediments collected from wetland pillars in the temperature simulation experiment (control: ambient temperature; low-warmed: ambient temperature +2.5 °C; high-warmed: ambient temperature +5.0 °C). The abbreviation of wetland sampling sites is identified on the *x*-axis, and the TOC content in the sediments is identified on the *y*-axis. Error bars represent the standard error of the mean of three parallel samples. Values within the same sampling site follow by the same small letters (a, b, c, d, e) are not significantly different at *p* = 0.05.

TOC gradations reached a scope of 2.66 g∙kg^−1^ to 7.73 g∙kg^−1^ and 3.12 g∙kg^−1^ to 8.60 g∙kg^−1^ for the control treatment and 2.5 °C warmed treatment, respectively (Figure 3). In the meantime, the 5.0 °C warmed treatment ranged a scope of 2.34 g∙kg^−1^ to 8.46 g∙kg^−1^. With the exception of the pillars from BHB, all the groups of TOC contents were increased under the warming condition, and warming treatment growing faster than the control group. In addition, the significance among the treatments was decreased gradually as time went on, and the flooding period was closely related to the increasing temperature.

**Figure 4:**
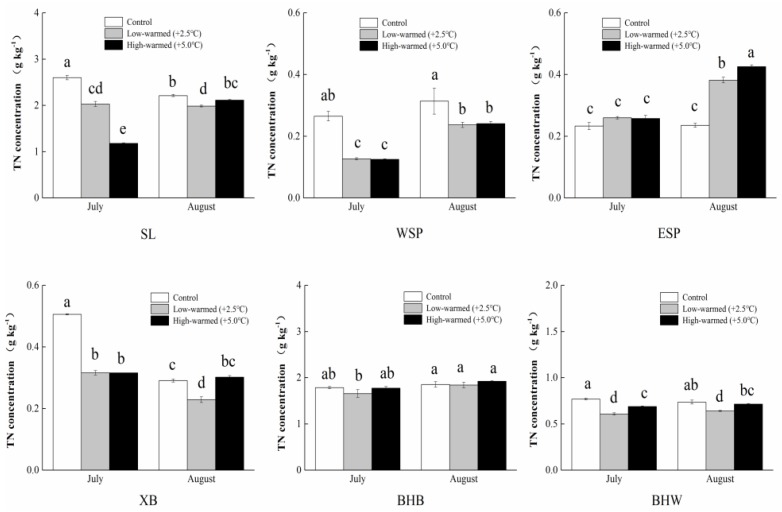
Statistical differences of total nitrogen (TN) in sediments collected from wetland pillars in the temperature simulation experiment (control: ambient temperature; low-warmed: ambient temperature +2.5 °C; high-warmed: ambient temperature +5.0 °C). The abbreviation of wetland sampling sites is identified on the *x*-axis, and the TN concentration in the sediments is identified on the *y*-axis. Error bars represent the standard error of the mean of three parallel samples. Values within the same sampling site follow by the same small letters (a, b, c, d, e) are not significantly different at *p* = 0.05.

**Figure 5:**
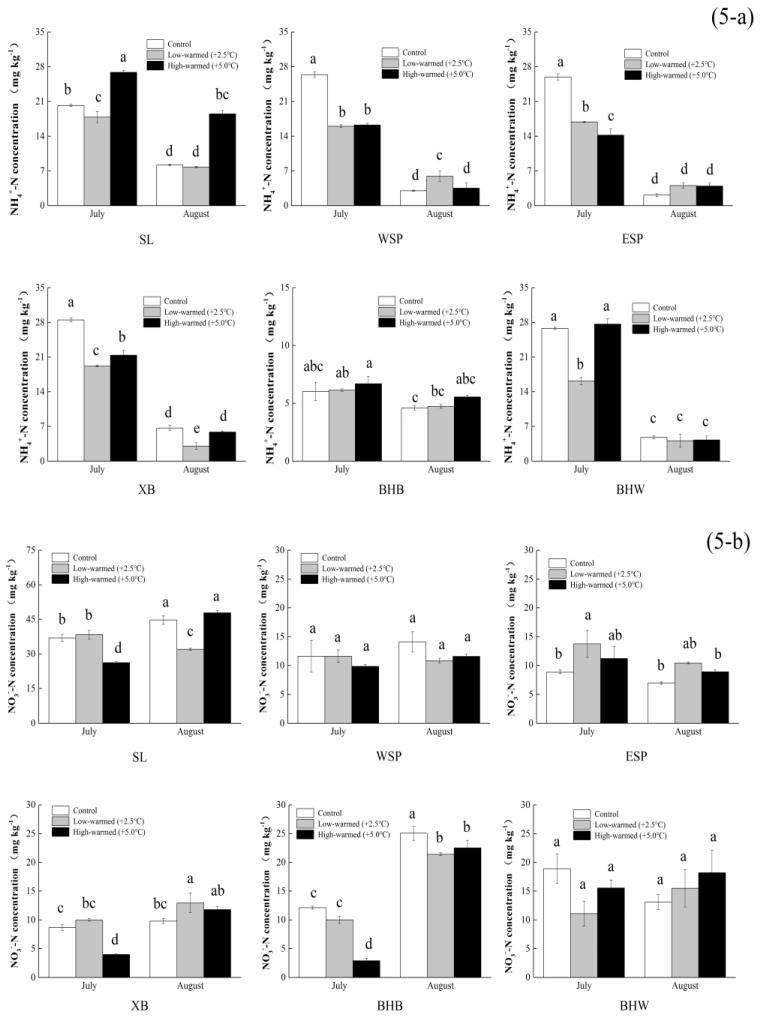
Statistical differences of NH_4_^+^-N (5-**a**) and NO_3_^−^-N (5-**b**) in sediments collected from wetland pillars in the temperature simulation experiment (control: ambient temperature; low-warmed: ambient temperature +2.5 °C; high-warmed: ambient temperature +5.0 °C). The abbreviation of wetland sampling sites is identified on the *x*-axis and the NH_4_^+^-N and NO_3_^−^-N concentrations in the sediments are identified on the *y*-axis. Error bars represent the standard error of the mean of three parallel samples. Means with the same small letters (a, b, c, d) are not significantly different at *p* = 0.05.

The levels of TOC in sediment were generally increased during the incubation. Meanwhile, the TN levels reached a scope of 0.23 g∙kg^−1^ to 2.60 g∙kg^−1^ for the incubation. The calculations show that TN levels in Swan Lake were greater than the other five sample plots (Figure 4). The variation trend of TN increased first and decreased afterwards, except in the ESP group. The levels of NH_4_^+^-N in the sediment for each pillar under the warmed treatment were generally lower than those of ambient treatment (*p* < 0.05) in the incubation, except the 5 °C-warmed treatment in SL and BHB, respectively (Figure 5a). Furthermore, the levels of NH_4_^+^-N in sediment generally decreased as time lapses, which had a special change of descending firstly, then ascending with the rising temperature in SL and XB in late summer. At the same time, the contents of NO_3_^−^-N increased first and decreased afterwards in ESP and XB with the elevation of temperature (Figure 5b). WSP and BHW had no significant difference in the incubation. The levels of NH_4_^+^-N and NO_3_^−^-N for the XB and BHB pillars had a contrary trend in incubation.

**Figure 6:**
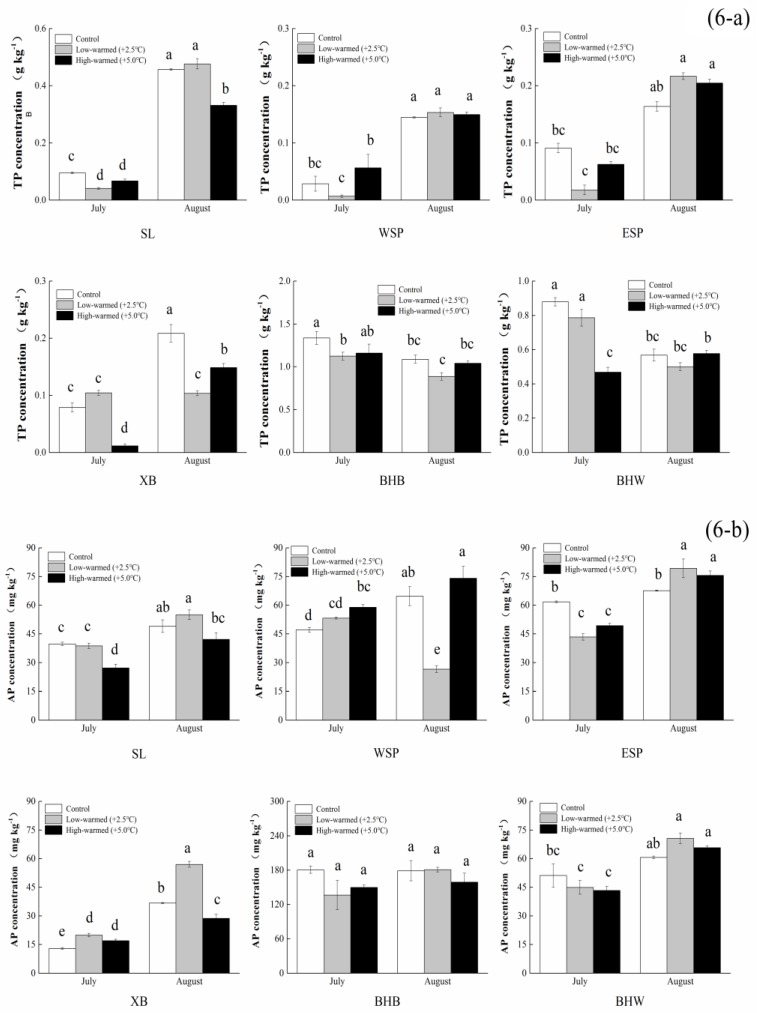
Statistical differences of Total Phosphorus (6-**a**) and Available Phosphorus (6-**b**) in sediments collected from wetland pillars in the temperature simulation experiment (control: ambient temperature; low-warmed: ambient temperature +2.5 °C; high-warmed: ambient temperature +5.0 °C). The abbreviation of wetland sampling sites is identified on the *x*-axis, and the TP and AP concentrations in the sediments are identified on the *y*-axis. Error bars represent the standard error of the mean of three parallel samples. Means with the same small letters (a, b, c, d, e) are not significantly different at *p* = 0.05.

We found that the TP contents in BHW and BHB with seasonal flooding periods decreased in warming as time went on. The data indicate that there was no significant difference in ESP and the latter part of BHW, BHB, and WSP (Figure 6a). The TP contents in BHB and BHW were decreased with the elongation of the incubation time. The statistical analysis exhibited that the TP contents in warmed pillars were generally lower than those of the control treatment in seasonal flooding periods of GIW. The contents of TP in Swan Lake were significantly increased 4.50–4.94 times, which increased greater than the other groups.

It can be determined that the influences of warming contributed to TN and TP discharging from the sediment into the water. The two gradient-warmed treatments showed remarkably declining levels of TN in SL samples, with decreases from 2.60 g∙kg^−1^ to 1.17 g∙kg^−1^, and 44.70 mg∙kg^−1^ to 31.97 mg∙kg^−1^ for NO_3_^−^-N. Similarly, TP levels under the warmed treatment were basically smaller than those of the control samples (*p* < 0.05). Under the control treatment, the AP levels ranged from 12.97 mg∙kg^−1^ (XB) to 180.66 mg∙kg^−1^ (BHB); meanwhile, those levels under the 2.5 °C warmed treatment ranged from 19.94 mg∙kg^−1^ (XB) to 181.00 mg∙kg^−1^ (BHB), and the 5.0 °C warmed treatment ranged from 17.03 mg∙kg^−1^ (XB) to 158.79 mg∙kg^−1^ (BHB), respectively (Figure 6b). The AP levels increased with time, except BHB and BHW, and XB increased faster than the other five wetland sites in temperature variation. The statistical analysis shows that the increased temperature prompted generally lower TN and TP levels in the warmed treatments than those of the controlled treatment.

### 3.2. Redundancy Analysis and Pearson Correlation of Biogeochemical Properties and Enzyme Activity in Sediment of Different Flooding Periods in GIW

Table 2 and Table 3 show the activity of the detected four enzymes, namely, sucrase, urease, alkaline phosphatase, and catalase in sediments from the top 10 cm of wetland pillars sampled from six sampling sites in June 2016. From these tables, we can see that warming caused a shift in the sediment physicochemical index and biogeochemical properties. However, no significant difference was found between the control and warmed treatments in the portion of the carbon, nitrogen, or phosphorus levels and enzyme activity. For this reason, it can be inferred that the elevation of ambient temperature generated an influence over the different flooding periods of GIWs’ sediments, though not completely. Pearson correlation analysis shows that SUC, URE, TN, and NO_3_^−^-N had significant negative correlation with pH and EC (Table 4). TOC was significant positively related to AP and TN, in the meantime, TP was significant positively related to AP and AKP; TN was positively related to NO_3_^−^-N, but the opposite was true with NH_4_^+^-N (*p* < 0.01). Furthermore, CAT has a significant negative correlation with NO_3_^−^-N, TOC, TN, TP, AP, and URE, but opposite to NH_4_^+^-N. Moreover, CAT and EC were significantly and positively related to pH. NH_4_^+^-N was correlated negatively with TN, TP, TOC, and AP (*p* < 0.01).

**Figure 7:**
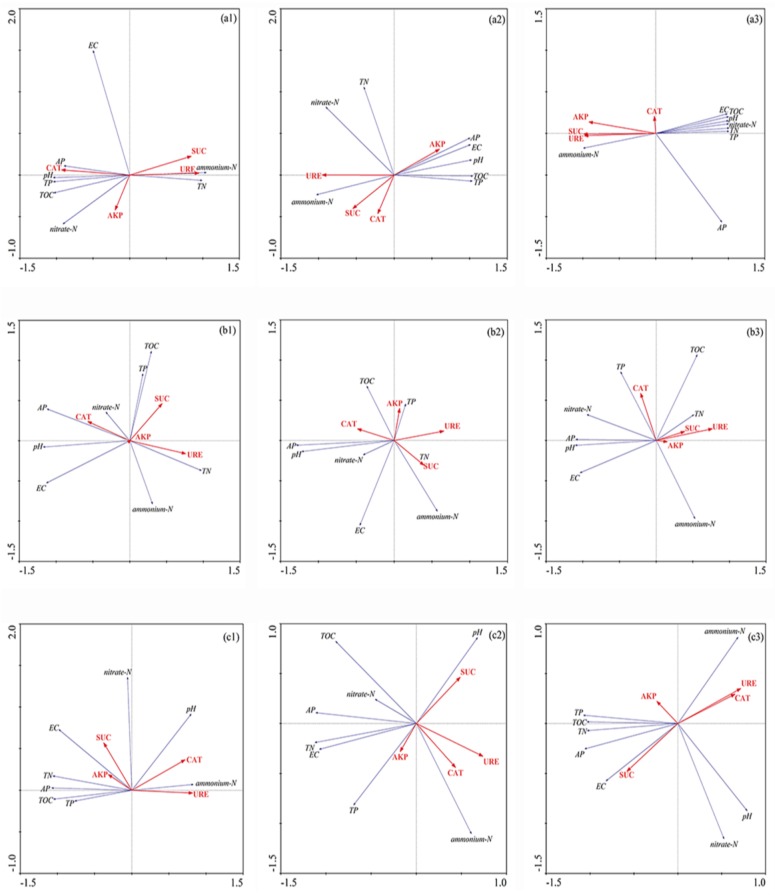
Redundancy analysis of sediment enzyme activities with its biogeochemical properties under the simulation experiment. TOC, total organic carbon; TN, total nitrogen; TP, total phosphorus; AP, available phosphorus; SUC, sucrase; URE, urease; AKP, alkaline phosphatase; CAT, catalase; pH, potential of hydrogen; EC, electrical conductivity; ammonium-N, ammonium nitrogen; nitrate-N, nitrate nitrogen; (**a1**) control treatment of GIW in permanent flooding period; (**a2**) 2.5 °C warmed treatment of GIW in a permanent flooding period; (**a3**) 5.0 °C warmed treatment of GIW in a permanent flooding period; (**b1**) control treatment of GIW in an inter-annual flooding period; (**b2**) 2.5 °C warmed treatment of GIW in an inter-annual flooding period; (**b3**) 5.0 °C warmed treatment of GIW in an inter-annual flooding period; (**c1**) control treatment of GIW in a seasonal flooding period; (**c2**) 2.5 °C warmed treatment of GIW in a seasonal flooding period; (**c3**) 5.0 °C warmed treatment of GIW in a seasonal flooding period.

The research findings show that TOC, TP, and pH had significant negative correlation with SUC, NH_4_^+^-N and URE in all three treatments of GIW with a permanent flooding period. SUC was negatively correlated with CAT in the ambient group, and a linear positive correlation existed between them in the 2.5 °C warmed treatment, but there was no obvious correlation between them in the 5.0 °C warmed treatment (Figure 7a1–a3). On the other hand, AP was positively correlated with NO_3_^−^-N and pH in GIW with an inter-annual flooding period, and all of them had significant negatively correlation with URE. TOC was significantly positively related to TN in the 5.0 °C warmed treatment, and the opposite was true in the 2.5 °C warmed treatment, but it was uncorrelated in the control group. Furthermore, AP was significant positively related to TN and EC in GIW with a seasonal flooding period. During the experiments, URE was significant negatively related with them all along. SUC was positively correlated with AKP in the control group, and we found a negative correlation between them in the 2.5 °C warmed treatment, but there was no correlation between them in the 5.0 °C warmed treatment, ultimately.

## 4. Discussion

### 4.1. Effects of Sediment Biogeochemical Characteristics Responding to Ambient Temperature Warming

Carbon serves as an energy medium and a metabolism storeroom [33,34] for phosphorus transformation in the water-sediment system of wetlands. The TOC content was increased significantly, but changed smoothly as time went on, except in BHB, with a lower water content (Figure 3), which possibly indicate a sharp increase of the microbial metabolism. It is observed that during the seasonal flooding period, the GIW’s amplitude of fluctuation is higher than the others in the level of TN. Similarly, these data fluctuations were smaller in carbon and phosphorus than nitrogen for warmed treatments in the process of incubation. A possible explanation is that the too frequently alternation of drying and wetting disturbed the microorganisms’ metabolic activity. Total nitrogen content in pillars was generally decreased with temperature increasing except ESP group possibly because the increased denitrification and volatilization of ammonia which released from sediment to overlying water. The values of NH_4_^+^-N and NO_3_^−^-N in warmed sediments (at 10 cm depth) in these pillars were commonly lower than those in the control treatment, probably through nitrogen decomposition, resulting in the dissolution of nitrogen for sediments in warmed pillars. The significant differences were found in carbon and nitrogen contents between the warmed and control treatments in a majority of the sampling events, separately. In other words, the intensity of chemical components released from upper layer sediment to overlying water and sublayer sediments could be quickly improved.

Furthermore, earlier studies showed that phosphates dissolved in sediment pore water were positively correlated with temperature (*p* < 0.01) for lake sediments [35,36,37] and vegetated buffer strips [38]. Similarly, the TP levels of sediment in the warmed treatments were lower than the controls. The shift in enzyme activity composition towards higher relative levels of chemical components in these warmed pillars could increase the utilization efficiency in sediment [39,40], which might explain the comparatively high contents of phosphorus and carbon found in the sediment under warming. These findings support the hypothesis that warming increased the potential of nutrient component release from the superstratum sediment to the overlying water or sublayer sediment, where the ecological role of the source sediment was more declared. Although much is known about the reaction substrate, reaction rate, reaction process, and controlling mechanisms, the changing process of new findings discovered in the last decade demonstrates that understanding and predicting the response in sediment to climate warming remains a formidable but exciting, and indeed “deliciously complex” task.

### 4.2. Effects of Sediment Enzyme Activities Responding to Ambient Temperature Warming 

Climate changes may alter soil microclimates and nutrient availability, ultimately modifying relationships between soil enzyme activity and soil environmental conditions. Massive investment has been made to restore wetlands, with the target of restoring the wetland ground water environment and building a harmonious natural ecology. Unfortunately, the present situation is serious and comes with escalating temperature and environmental degradation. It is well known that global warming will have an active effect on enzyme activities and environmental protection. Instead, from the current research status, we can comprehend that warming significantly restrained urease activity, the content of total nitrogen, and NH_4_^+^-N in sediment. It was clear that the carbon, as well as nitrogen, cycles were modified when the isolated wetland sediment was warmed with a temperature control device since these enzymatic activities were stimulated during the period of warming. Sediment sucrase is significant for circulation of organic matter, and inhibition and activation effects may have an influence on the mineralization of relevant nutrients [41]. The change of enzyme activities is likely related to the high content of organic matter [42,43], and temperature fluctuation shifted the metabolic activity in the sediment.

Some studies have proved that experimental warming could promote urease activity, but it’s not necessarily the case [40,44]. Besides, the availability of the sediment enzyme substrate may be weakened by the reduction of sediment water content [45]. Carbon availability increases the nitrogen-cycling enzyme activity and, in the meantime, carbon-cycling enzyme activity increased with inorganic nitrogen availability. Differences in organic agroecosystem management have strongly influenced soil nutrients and potential enzyme activity [46]. Development of better indicators such as soluble organic nitrogen of wetland sediment functions in an ecological system may help managers evaluate and formulate management options that improve the purifying environment capacity of the sediment.

Previous research has revealed that warming frequently promotes phosphatase activity [44,47,48,49]. Warming increased the activities of alkaline phosphatase activity (22%) in the spring in a shrubland soil field experiment within a one-year period [44,50], showing that pH was a leading factor for phosphatase activity in wetland sediments. However, we did not find the correlation explained by the meagre variation in pH. The intensities of phosphorus released from sediment to both pore-water and overlying water are improved under warming conditions [18,35,51]. The phosphorus level changes in warmed treatments was contrary to that of phosphatase activity. Phosphorus balance between sediment and water was vulnerable to elevated temperatures in the isolated wetland of the fragile grassland ecosystem. In addition, as an intracellular enzyme, catalase decomposes hydrogen peroxide into molecular oxygen and water. In this study, catalase activity showed no significant variation during the period of warming. It seems that the temperature warming exercised no significant influence on the catalase activity. Moreover, pH, TP, and TOC showed a closely-linked with SUC, URE, and NH_4_^+^-N in GIW with a permanent flooding period by the analysis of RDA. In addition, pH, AP, and NO_3_^−^-N were closely linked with SUC and URE in GIW with an inter-annual flooding period. However, AP, TN, and EC were significantly negatively correlated with URE in GIW with a seasonal flooding period.

Most of the variation in enzyme activity could be partly explained by soil characteristics related to nutrient availability; just like sediment features to be affected by various factors, including sediment type and composition of grain diameters. The unconstrained uncertainties suggest that we still cannot clarified the response of biochemistry in sediment to climate warming completely, we probably cannot properly parameterize all of the various rates and processes, and we therefore can develop collaboration research and subject crossing in the long run.

## 5. Conclusions

The results of our study show that warming facilitated a massive fluctuation of nutritional ingredients from wetland sediment to water, and obstructed the natural subsidence of sediment, but reinforced the character of the ecological source. The contents of TOC, TN, and TP in warmed treatments showed a rough balance or a slightly decrease than the control treatment, and NH_4_^+^-N suffered a larger decline under warming conditions, except the 5.0 °C-warmed treatment of SL. Furthermore, the seasonal flooding period of GIW exhibited a smaller variation than the others with respect to sediment biogeochemical properties.

On the other hand, warming reduced the activity of urease, but promoted the activity of alkaline phosphatase and sucrase. Meanwhile, catalase had no significant variation under the warming condition. Redundancy analysis showed that sucrase, urease, available phosphorus and pH were the major correlated factors under the warming conditions in our research scope. Total carbon, total nitrogen, sucrase, catalase, and alkaline phosphatase were the principal reference factors to reflect the ambient temperature variations. Our study results indirectly show that nutrient compositions and enzyme activities in GIW ecosystems could be reconstructed under a warming influence. Nonetheless, the long-range effect of warming on physicochemical properties and enzyme activity in GIWs still demands more comprehensive, thorough, investigation and survey to ascertain the variation mechanisms.

## Figures and Tables

**Table 1:** Original characteristics of GIWs’ sediment in temperature simulation experiments under different flooding periods in the Hulunbuir Grassland.

Parameters	SL	WSP	ESP	XB	BHW	BHB
pH	8.67 ± 0.01 ^e^	10.27 ± 0.04 ^c^	10.44 ± 0.01 ^b^	9.71 ± 0.03 ^d^	10.61 ± 0.02 ^a^	10.31 ± 0.01 ^c^
EC (μS·cm^−1^)	328.33 ± 3.75 ^e^	2320.00 ± 26.45 ^b^	3053.33 ± 114.64 ^a^	453.33 ± 8.83 ^e^	1507.00 ± 4.61 ^d^	2110.00 ± 11.54 ^c^
TOC (g·kg^−1^)	5.925 ± 0.141 ^b^	3.523 ± 0.131 ^c^	2.971 ± 0.215 ^d^	3.976 ± 0.083 ^c^	2.663 ± 0.18 ^d^	7.194 ± 0.172 ^a^
TN (g·kg^−1^)	2.602 ± 0.049 ^a^	0.265 ± 0.016 ^e^	0.233 ± 0.011 ^e^	0.506 ± 0.002 ^d^	0.768 ± 0.013 ^c^	1.786 ± 0.022 ^b^
NH_4_^+^-N (mg kg^−1^)	20.194 ± 0.216 ^c^	26.398 ± 0.59 ^b^	25.938 ± 0.612 ^b^	28.52 ± 0.423 ^a^	26.802 ± 0.248 ^b^	6.02 ± 0.804 ^d^
NO_3_^−^-N (mg·kg^−1^)	36.96 ± 1.572 ^a^	11.606 ± 2.748 ^c^	8.879 ± 0.306 ^c^	8.67 ± 0.499 ^c^	18.922 ± 2.555 ^b^	12.122 ± 0.258 ^c^
TP (g·kg^−1^)	0.095 ± 0.003 ^c^	0.029 ± 0.013 ^c^	0.091 ± 0.008 ^c^	0.079 ± 0.008 ^c^	0.879 ± 0.024 ^b^	1.337 ± 0.075 ^a^
AP (mg·kg^−1^)	39.767 ± 0.873 ^c^	47.163 ± 1.221 ^c^	61.761 ± 0.413 ^b^	12.978 ± 0.464 ^d^	51.206 ± 6.172 ^b,c^	180.662 ± 6.513 ^a^
SUC (mg glucose·g^−1^ 24 h^−1^)	10.371 ± 3.301 ^a^	0.138 ± 0.026 ^b^	0.168 ± 0.029 ^b^	3.478 ± 0.069 ^b^	1.596 ± 0.276 ^b^	0.451 ± 0.043 ^b^
URE (mg NH_4_^+^-N·g^−1^·24 h^−1^)	1.424 ± 0.002 ^a^	0.087 ± 0.001 ^d^	0.098 ± 0.011 ^d^	0.567 ± 0.052 ^c^	0.649 ± 0.002 ^b^	0.155 ± 0.021 ^d^
AKP (mg phenol·g^−1^ 24 h^−1^)	0.041 ± 0.005 ^a^	0.036 ± 0.002 ^a^	0.061 ± 0.017 ^a^	0.035 ± 0.006 ^a^	0.065 ± 0.006 ^a^	0.066 ± 0.013 ^a^
CAT (mg H_2_O_2_·g^−1^·20 min^−1^)	0.117 ± 0.001 ^c^	0.305 ± 0.022 ^a^	0.299 ± 0.012 ^a^	0.242 ± 0.024 ^b^	0.229 ± 0.002 ^b^	0.101 ± 0.004 ^c^

Note: Values are the mean ± standard error; a, b, c, d, e values within the same line not followed by the same letter indicate statistical differences (*p* < 0.05). SL, Swan Lake; WSP, West Spectacles Pond; ESP, East Spectacles Pond; XB, Xibo Bridge; BHW, Bei Hui White; BHB, Bei Hui Black; pH, potential of hydrogen; EC, electrical conductivity; TOC, total organic carbon; TN, total nitrogen; NH_4_^+^-N, ammonium nitrogen; NO_3_^-^-N, nitrate nitrogen; TP, total phosphorus; AP, available phosphorus; SUC, sucrase; URE, urease; AKP, alkaline phosphatase; CAT, catalase.

**Table 2:** Enzyme activity of the six GIWs in different flooding periods after one month of incubation.

Sample Site	SUC (mg Glucose g^−1^·24 h^−1^)			URE (mg NH_4_^+^-N g^−1^ 24 h^−1^)			AKP (mg Phenol·g^−1^ 24 h^−1^)			CAT (mg H_2_O_2_ g^−1^ 20 min^−1^)		
	Control	Low Warmed	High Warmed	Control	Low Warmed	High Warmed	Control	Low Warmed	High Warmed	Control	Low Warmed	High Warmed
SL	9.371 ± 1.266	18.687 ± 1.779	7.219 ± 0.063	1.424 ± 0.002	1.238 ± 0.008	1.073 ± 0.032	0.041 ± 0.005	0.026 ± 0.007	0.118 ± 0.013	0.117 ± 0.012	0.161 ± 0.006	0.218 ± 0.002
WSP	0.158 ± 0.014	0.128 ± 0.027	0.337 ± 0.005	0.087 ± 0.001	0.083 ± 0.015	0.039 ± 0.002	0.036 ± 0.002	0.047 ± 0.013	0.036 ± 0.003	0.305 ± 0.022	0.347 ± 0.019	0.288 ± 0.021
ESP	0.141 ± 0.009	2.511 ± 0.252	1.024 ± 0.067	0.098 ± 0.011	0.062 ± 0.004	0.056 ± 0.005	0.061 ± 0.017	0.065 ± 0.004	0.061 ± 0.025	0.299 ± 0.012	0.244 ± 0.011	0.259 ± 0.005
XB	3.478 ± 0.069	1.511 ± 0.077	3.491 ± 0.114	0.567 ± 0.052	0.422 ± 0.012	0.317 ± 0.015	0.035 ± 0.006	0.058 ± 0.005	0.055 ± 0.026	0.242 ± 0.024	0.065 ± 0.032	0.286 ± 0.019
BHB	0.484 ± 0.021	0.736 ± 0.026	3.225 ± 0.145	0.155 ± 0.021	0.166 ± 0.008	0.215 ± 0.021	0.066 ± 0.013	0.049 ± 0.005	0.072 ± 0.013	0.101 ± 0.004	0.164 ± 0.023	0.155 ± 0.007
BHW	1.596 ± 0.276	0.602 ± 0.028	0.503 ± 0.022	0.649 ± 0.002	0.811 ± 0.049	0.648 ± 0.029	0.065 ± 0.006	0.043 ± 0.008	0.083 ± 0.012	0.229 ± 0.002	0.217 ± 0.011	0.893 ± 0.003

Note: Values are the mean ± standard error.

**Table 3:** Enzyme activity of the six GIWs in different flooding periods after two months of incubation.

Sample Site	SUC (mg Glucose·g^−1^ 24 h^−1^)			URE (mg NH_4_^+^-N·g^−1^ 24 h^−1^)			AKP (mg Phenol·g^−1^ 24 h^−1^)			CAT (mg H_2_O_2_·g^−1^ 20 min^−1^)		
	Control	Low Warmed	High Warmed	Control	Low Warmed	High Warmed	Control	Low Warmed	High Warmed	Control	Low Warmed	High Warmed
SW	4.079 ± 0.103	16.112 ± 0.131	0.515 ± 0.036	0.411 ± 0.016	0.332 ± 0.005	0.606 ± 0.009	0.045 ± 0.007	0.041 ± 0.005	0.038 ± 0.010	0.202 ± 0.011	0.158 ± 0.004	0.215 ± 0.051
WSP	0.145 ± 0.011	1.995 ± 0.171	3.142 ± 0.134	0.107 ± 0.021	0.172 ± 0.007	0.109 ± 0.005	0.029 ± 0.008	0.108 ± 0.021	0.036 ± 0.002	0.363 ± 0.008	0.342 ± 0.008	0.361 ± 0.017
ESP	0.125 ± 0.016	0.694 ± 0.029	1.291 ± 0.048	0.163 ± 0.005	0.084 ± 0.005	0.104 ± 0.002	0.032 ± 0.005	0.049 ± 0.005	0.065 ± 0.003	0.314 ± 0.005	0.255 ± 0.022	0.307 ± 0.024
XB	18.472 ± 0.245	0.138 ± 0.009	1.455 ± 0.117	0.241 ± 0.016	0.116 ± 0.002	0.185 ± 0.008	0.059 ± 0.012	0.112 ± 0.017	0.046 ± 0.006	0.282 ± 0.012	0.368 ± 0.002	0.342 ± 0.023
BHB	6.115 ± 0.484	0.127 ± 0.007	7.542 ± 0.176	0.089 ± 0.003	0.188 ± 0.001	0.072 ± 0.001	0.072 ± 0.013	0.049 ± 0.005	0.086 ± 0.008	0.168 ± 0.006	0.169 ± 0.006	0.161 ± 0.015
BHW	0.405 ± 0.054	1.323 ± 0.024	4.006 ± 0.078	0.311 ± 0.018	0.329 ± 0.002	0.208 ± 0.001	0.038 ± 0.008	0.034 ± 0.007	0.025 ± 0.006	0.172 ± 0.005	0.155 ± 0.004	0.112 ± 0.008

Note: Values are the mean ± standard error.

**Table 4:** Pearson correlation matrix for sediment variables sampled under simulated temperature fields.

	NO_3_^−^N (mg·kg^−1^)	TN (g·kg^−1^)	TP (g·kg^−1^)	SUC (mg·g^−1^·24 h^−1^)	URE (mg·g^−1^·24 h^−1^)	CAT (mg·g^−1^·20 min^−1^)	TOC (g·kg^−1^)	AKP (mg·g^−1^·24 h^−1^)	AP (mg·kg^−1^)	pH	EC (μS·cm^−1^)
NH_4_^+^-N (mg·kg^−1^)	–0.112	–0.638 **	–0.717 **	0.002	0.069	0.765 **	–0.886 **	–0.360	–0.919 **	0.046	–0.109
NO_3_^−^-N (mg·kg^−1^)		0.790 **	–0.076	0.735 **	0.878 **	–0.554 *	0.316	–0.076	–0.156	–0.743 **	–0.564 *
TN (g·kg^−1^)			0.302	0.649 **	0.706 **	–0.903 **	0.804 **	0.037	0.334	–0.700 **	–0.519 *
TP (g·kg^−1^)				–0.281	–0.194	–0.545 *	0.441	0.527 *	0.814 **	0.420	0.156
SUC (mg glucose·g^−1^·24 h^−1^)					0.847 **	–0.454	0.311	–0.309	–0.326	–0.839 **	–0.708 **
URE (mg NH4+-N g^−1^ 24 h^−1^)						–0.500 *	0.232	–0.194	–0.393	–0.834 **	–0.829 **
CAT (mg H_2_O_2_·g^−1^·20 min^−1^)							–0.857 **	–0.192	–0.529 *	0.480 *	0.448
TOC (g kg^−1^)								0.085	0.656 **	–0.452	–0.257
AKP (mg phenol·g^−1^ 24 h^−1^)									0.490 *	0.410	0.376
AP (mg·kg^−1^)										0.327	0.421
pH											0.759 **

Note: ** Correlation is significant at the 0.01 level (two-tailed); * correlation is significant at the 0.05 level (two-tailed).
